# Application of One-Dimensional Nanomaterials in Catalysis at the Single-Molecule and Single-Particle Scale

**DOI:** 10.3389/fchem.2021.812287

**Published:** 2021-12-17

**Authors:** Saisai Yuan, Qitao Zhang

**Affiliations:** ^1^ School of Environmental and Chemical Engineering, Jiangsu University of Science and Technology, Zhenjiang, China; ^2^ International Collaborative Laboratory of 2D Materials for Optoelectronics Science and Technology of Ministry of Education, Institute of Microscale Optoelectronics, Shenzhen University, Shenzhen, China

**Keywords:** 1D nanomaterials, photocatalysis, electrocatalysis, single-particle, single-molecule

## Abstract

The morphology of nanomaterials has a great influence on the catalytic performance. One-dimensional (1D) nanomaterials have been widely used in the field of catalysis due to their unique linear morphology with large specific surface area, high electron-hole separation efficiency, strong light absorption capacity, plentiful exposed active sites, and so on. In this review, we summarized the recent progress of 1D nanomaterials by focusing on the applications in photocatalysis and electrocatalysis. We highlighted the advanced characterization techniques, such as scanning tunneling microscopy (STM), atomic force microscopy (AFM), surface photovoltage microscopy (SPVM), single-molecule fluorescence microscopy (SMFM), and a variety of combined characterization methods, which have been used to identify the catalytic action of active sites and reveal the mechanism of 1D nanomaterials. Finally, the challenges and future directions of the research on the catalytic mechanism of single-particle 1D nanomaterials are prospected. To our best knowledge, there is no review on the application of single-molecule or single-particle characterization technology to 1D nanomaterial catalysis at present. This review provides a systematic introduction to the frontier field and opens the way for the 1D nanomaterial catalysis.

## Introduction

One-dimensional (1D) nanomaterial is a special structure of substance in nanometer scale and is a key branch of nanomaterial systems. It has been widely developed and been used in many fields due to its unique linear morphology with large specific surface area, high electron-hole separation efficiency, strong light absorption capacity, plentiful exposed active sites, and other characteristics (Linares et al., 2014). In general, 1D nanostructures have a high aspect ratio, with the diameters ranging from 1 to 100 nm (Xia et al., 2003), and their morphologies can be wired (Tian et al., 2007), tubular (Patil et al., 2017), rod-like (Liu et al., 2016a), fibrous (Hou et al., 2016), or banded (Sun et al., 2016). The preparation of 1D nanostructures mainly includes hydrothermal method (Byrappa and Adschiri, 2007), vapor deposition method (George, 2010), sol–gel method (Sui and Charpentier, 2012), template method (Ghahremaninezhad and Dolati, 2009), and electrospinning method (Li and Xia, 2004). Therefore, based on the characteristics of 1D nanomaterials, it has significant advantages in light capture, electron and ion transmission, and mass loading and diffusion and has an important application prospect in the field of mesoscopic, energy storage and conversion (Mai et al., 2014; Cao et al., 2019), nano-optoelectronic devices (Li et al., 2013), photocatalysis (Liu et al., 2010), and electrocatalysis (Gao et al., 2018).

**FIGURE 1:**
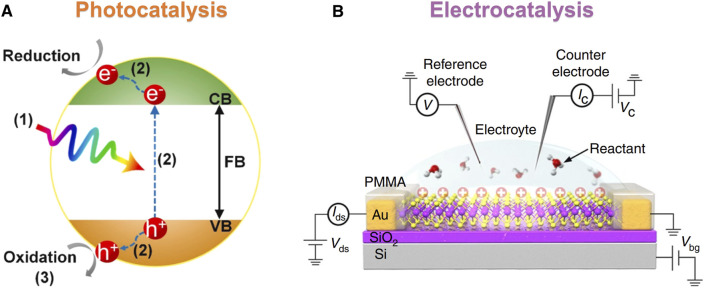
Schematic diagram of the principles of photocatalysis and electrocatalysis. **(A)** The typical photocatalytic process goes through three steps (Zhu et al., 2021). **(B)** Schematic diagram of electrocatalytic measurement (He et al., 2019). Reproduced from Zhu et al. (2021) with the permission of the Elsevier. Reproduced from He et al. (2019) with the permission of Springer Nature.

Among these applications, photocatalysis and electrocatalysis are of great interest to us in this review. Heterogeneous surface catalysis had been used in various catalytic reactions and had led to extensive exploration of the catalytic activity of metallic and nonmetallic surface sites (Wang et al., 2014; Low et al., 2017). The principles of photocatalysis are shown in Figure 1A, and the charge generated by light needed to be effectively separated from the surface of the photocatalytic materials for the catalytic reaction. The catalytic efficiency mainly depended on the charge separation (Zhang et al., 2018), light absorption capacity (Yuan et al., 2017; Yuán et al., 2018), and specific surface area (Yuan et al., 2014; Yuan et al., 2018) of the catalyst. Fast charge separation and slow recombination were beneficial to the formation of more carriers on the catalyst surface and excellent catalytic performance. At the same time, the response to visible light could be enhanced by tailoring the bandgap structure of the semiconductor, which generated more photogenerated charge pairs and further improved the rate of photocatalysts. For electrocatalysis, as shown in Figure 1B, it was a catalysis that accelerates the charge transfer at the interface between electrodes and electrolytes by external applied voltage. The understanding of the reaction mechanism of electrocatalysis is important for the design and development of electrocatalytic materials. The electrocatalytic performance was affected by the structure–activity relationship, the distribution of active sites, and the surface process of electrocatalytic reaction.

**FIGURE 2:**
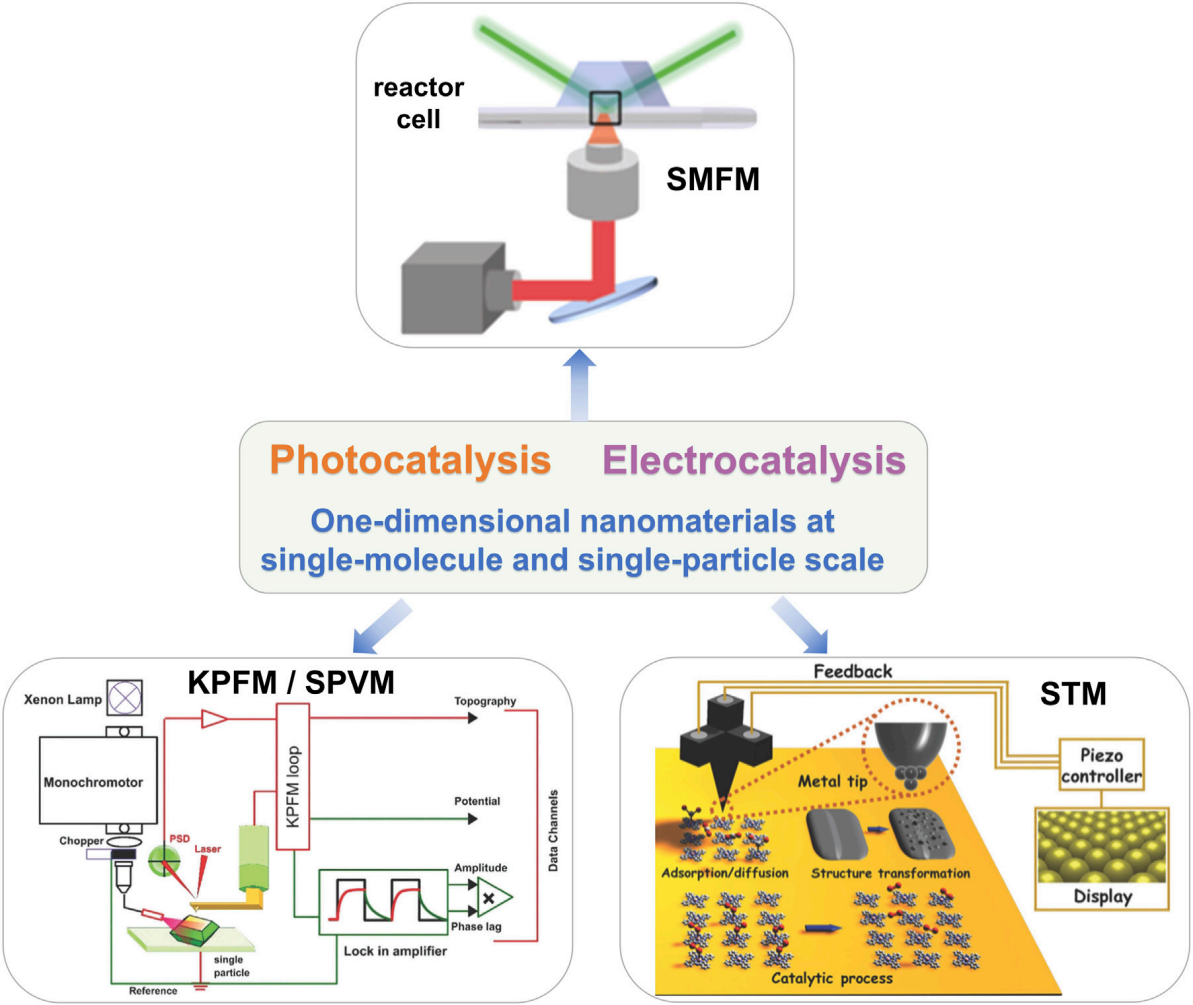
The techniques for the investigation of 1D-nanomaterial catalysis at single-molecule and single-particle scale. The SMFM (Andoy et al., 2013), KPFM/SPVM (Chen et al., 2018), and STM (Wang et al., 2021) characterization tools were introduced in this review. Reproduced from Andoy et al. (2013) with the permission of the American Chemical Society. Reproduced from Chen et al. (2018) with the permission of the Royal Society of Chemistry. Reproduced from Wang et al. (2021) with permission of the Royal Society of Chemistry.

No matter in the field of photocatalysis or electrocatalysis, because of the complexity of the catalytic system, it is necessary to comprehensively characterize the catalytic process and deeply reveal the mechanism of the catalytic process, which is crucial to further develop the design of functional catalytic materials and further improve the catalytic performance. As we know, the catalytic reactions always take place on the surface of the catalyst, and the finer we disperse the same mass of material, the more atoms on the surface are exposed, resulting in the catalyst with a larger specific surface (Liu et al., 2016b; Chao et al., 2021). Reducing the size of the catalyst to a single particle can not only increase the effective utilization rate of the catalyst but also greatly improve its catalytic activity. In addition, compared with bulk catalysts, single-molecule or single-particle catalysts have a simple structure, have a clear definition of active sites, and avoid multi-pathway reactions in the catalytic process, which has a great advantage in the investigation of reaction mechanisms. Up to now, many surface characterization techniques have been used to investigate the catalytic process with good spatial, chemical, and time resolution and have made great contributions to the understanding of the catalytic process. However, these traditional characterization techniques only obtained the average information of many catalytic particles and aggregates. The morphology, structure, crystal plane, and composition of a single catalytic particle strongly affected the overall performance of the catalyst (Tachikawa et al., 2011). It is an ideal strategy to directly apply characterization techniques to the real complex surface catalytic systems, especially to study the surface structure of the catalyst and the surface process of the catalytic reaction at the single-molecule scale. The development of advanced techniques provides the opportunities to study the catalytic mechanism at single-molecule and single-particle scale. As shown in Figure 2, scanning tunneling microscopy (STM), atomic force microscopy (AFM), surface photovoltage microscopy (SPVM), single-molecule fluorescence microscopy (SMFM), and so on, were of benefit to identify the catalytic action of active sites and reveal the mechanism of complex catalytic systems and further promote the design and development of novel catalysts (Novo et al., 2008; Tang et al., 2011).

In this review, we summarized the recent progress of 1D nanomaterials by focusing on the applications in photocatalysis and electrocatalysis. We highlighted the advanced characterization techniques, including STM, AFM, SPVM, SMFM, and a variety of combined characterization methods, which have been used to study the catalytic mechanism of 1D nanomaterials. The 1D-nanostructured materials that can be applied to catalysis in the form of single molecule and single particle were summarized as three types: 1) monitoring of catalytic reactions on individual particles using the SMFM technique; 2) visualization of photogenerated charge distribution of a single particle using the kelvin probe force microscopy (KPFM)/SPVM technique; and 3) morphological analysis of single particles and single atoms using STM and AFM techniques. Finally, the challenges and future directions of the research on the catalytic mechanism of single-particle 1D nanomaterials have been prospected. To our best knowledge, there is no review on the application of single-particle or single-molecule characterization technology to 1D nanomaterial catalysis at present. This review provides a systematic introduction to this frontier field and opens the way for 1D nanomaterial catalysis.

### Photocatalysis

Among nanomaterials, 1D-nanostructured materials with controllable morphology, large surface area, and more exposed active sites have attracted wide attention in photocatalysis. As a typical photocatalyst, the TiO_2_-nanostructured catalyst was a wide-gap semiconductor with excellent physical and chemical properties (Choi et al., 2010; Cao et al., 2018). The 1D TiO_2_ nanofiber structure photocatalyst prepared through the foam-assisted electrospinning method by Wu had a complete mesoporous channel and uniform pore structure (Hou et al., 2014), which provided a large number of active sites for adsorption reactants and an efficient route for gas transport. Thus, it was favorable for the precipitation rate of photocatalytic hydrogen production. In addition, the TiO_2_ catalyst with a 1D nanofiber structure was more stable than commercial P25 products due to the nanofiber structure inhibiting the aggregation of nanomaterials and stabilizing its morphology. However, it was not well understood how to precisely locate the catalytic active site in 1D-nanostructured catalysts and to what extent to drive charge separation in the photocatalytic reactions. The lack of understanding of these issues will become the bottleneck for further improvement of the photocatalytic efficiency. Therefore, the exploration of the 1D photocatalyst at single-particle and single-molecule scale is urgent for the investigation of microscopic mechanisms.

To solve these problems, it was important to directly image individual photocatalytic particles or photocatalyst interfaces. Super-resolution fluorescence microscopy techniques open up new prospects for visualization of individual catalytic events and localize the catalytic activity sites at the single nanocatalyst level (Roeffaers et al., 2006; Xu et al., 2008). The Au@mSiO_2_ materials formed by encapsulation of single gold nanorods in mesoporous silica shells were catalyzed for quantitative imaging using super-resolution fluorescence microscopy techniques (Zhou et al., 2012). These nanorods scatter laser light and emit it, making them easy to identify under an optical microscope. The relationship between the fluorescence intensity of individual nanorods and the time trajectory showed stochastic intensity bursts over the constant nanorods. Each stochastic intensity burst was marked by the catalytic production of fluorescent molecules. Simultaneously, the fluorescent molecules can be located from the fluorescence image by Gaussian fitting. This technique can image a single molecule and locate the active site, allowing the researcher to discover the complex patterns of catalytic activity on individual nanorods. The nanorods with the same surface composition on both sides and ends should have the same catalytic activity in conventional view; however, the catalytic reaction rate in the same surface was not constant as revealed in this work and presented a gradient distribution along the center to both ends of the nanorods. This could be reasonably explained by the defect density distribution on the nanorod surfaces in crystal growth.

With the development of probe microscope techniques, the charge carriers can be visualized *via* the spatially resolved SPV technique. For example, the function and properties of 1D TiO_2_ can be modified by gold nanoparticles (Au NPs) with SPR (Wang et al., 2017). The plasma water oxidation could be realized on the surface of Au/TiO_2_ under the central wavelength of about 550-nm absorption light. In this process, there was an obvious ring at the interface between Au NPs and TiO_2_ in the SPVM technique, and the surface photovoltage (SPV) was increased at the interface through the detailed analysis. There were two possible reasons for this phenomenon. One was that the holes generated by plasma resonance tended to accumulate at the interface between Au NPs and TiO_2_, and the other was that the Schottky barrier at the interface promoted the transfer of hot electrons to TiO_2_ and prevented its recombination. This discovery not only provided clear evidence for the distribution of hot holes but also confirmed that the Schottky barrier played an important role in promoting charge separation and stabilizing hot holes.

The investigation of 1D photocatalysts on single-molecule and single-particle scale will be helpful to understand the microscopic reaction and the charge separation, which will provide inspiration for us to design efficient photocatalysts.

### Electrocatalysis

Noble metal catalysts have attracted wide attention for their excellent catalytic performance, but the high price and limited reserves prevent their large-scale use. In the present studies, researchers reduced or avoided the use of noble metals through loading and alloying strategies or development of non-noble metal catalysts (Seh et al., 2017). Gao et al. chemically synthesized Pd/CeO_2_/C 1D nanostructure hybrid materials by the hydrothermal method (Gao et al., 2018). Compared to pure Pd/C, CeO_2_/C, and a physical mixture of Pd/C and CeO_2_/C, the chemically synthesized Pd/CeO_2_/C 1D nanostructure showed excellent catalytic activity and stability in the electrocatalytic hydrogen evolution reaction (HER). The main reason was that the chemical synthesis method had stronger interface coupling ability between Pd and CeO_2_ than the physical mixture method. However, this work did not explore the properties of the electrocatalyst interface at the single-particle scale, and the catalytic reaction mechanism was a little vague.

To further distinguish the reactivity of different catalyst surfaces and sites, the most direct method is to detect catalytic reactions *in situ* with spatial resolution on a single catalyst particle. The super-resolution imaging technique, such as STM, AFM, and SMFM, has gained considerable recognition at the level of a single molecule/particle, because of their ability to reveal and detect the dynamic system, providing detailed information about the individual molecule reaction, adsorption, and desorption process, as well as the distribution of active sites of single-particle catalysis (Carbonio et al., 2014). Other atom-resolved microscopes with distinctive features were also used in electrocatalytic reaction. AFM can provide morphological information of conductive and non-conductive samples. The degradation process of the Pt–Ni alloy catalyst was studied by *in situ* electrochemical atomic force microscopy (EC-AFM) during the accelerated durability test (ADT), and the coarsening process of the catalyst surface was observed during potential cycles (Khalakhan et al., 2017).

Among the non-noble metal electrocatalysts, carbon nanotubes are the most famous type of 1D nanomaterials. The SMFM imaging technique is also one of the advanced methods to characterize single molecules (Platnich et al., 2019; Bacic et al., 2020; Hao et al., 2020). Xu et al. studied the electrocatalytic fluorescence reaction by purified single-walled carbon nanotubes (SWNTs) using the SMFM imaging technique at the single-molecule level (Xu et al., 2009). In the electrocatalytic fluorescence reaction, the SMFM imaging technique had the ability of super-resolution localization of single fluorescence products and of determining the catalytic active sites on SWNTs, indicating that the fluorescence bursts of individual products were attributed to the electrocatalytic reduction of the reactants on SWNTs.

In short, the introduction of advanced characterization techniques plays an important role in revealing the reaction mechanism and determining the catalytic active sites. It also provides a powerful path for the design of novel electrocatalytic materials in the future.

## Conclusion and Perspectives

In this review, recent progress of 1D nanomaterials on the applications in photocatalysis and electrocatalysis was summarized. The single-molecule and single-particle investigation of 1D nanomaterials was reviewed, which had proved to be capable of improving the understanding of complex processes in the field of catalysis and gave deep insights into optimizing the performance of catalytic systems. This review provides a systematic introduction to the frontier field and opens the way for 1D nanomaterial catalysis.

To data, the properties of photocatalytic or electrocatalytic process are obtained from one individual particle. However, the preparation of single-particle nanomaterials still faces significant challenges due to the subtle effects of size, shape, and structural defects. At the same time, the present characterization technique is also very dependent on the external environment, which often causes randomness and volatility of data at the single-molecule and single-particle scale. For future, the first step is to improve the preparation methods of single-particle nanomaterials. Secondly, it is necessary to optimize the stability of advanced characterization techniques and unify standardized measurement. The results obtained from different research groups may be affected by the instrument settings and measurement environments. Finally, to enhance the efficiency of testing, high-speed acquisition and automated data analysis are required, and mathematical tools such as big data processing and machine learning will shine in these fields.
