# Corrigendum: Application of One-Dimensional Nanomaterials in Catalysis at the Single-Molecule and Single-Particle Scale

**DOI:** 10.3389/fchem.2022.920121

**Published:** 2022-05-03

**Authors:** Saisai Yuan, Qitao Zhang

**Affiliations:** ^1^ School of Environmental and Chemical Engineering, Jiangsu University of Science and Technology, Zhenjiang, China; ^2^ International Collaborative Laboratory of 2D Materials for Optoelectronics Science and Technology of Ministry of Education, Institute of Microscale Optoelectronics, Shenzhen University, Shenzhen, China

**Keywords:** 1D nanomaterials, photocatalysis, electrocatalysis, single-particle, single-molecule

In the original article, there was a mistake in the order of authors and, additionally, two authors were listed erroneously. The correct author list appears above.

In the original article, there was an error in affiliation 1, and affiliation 3 was included erroneously. The correct affiliations appears above.

The authors apologize for these errors and state that this does not change the scientific conclusions of the article in any way. The original article has been updated.

